# Evaluating Sedation Strategies for Magnetic Resonance Imaging: A Comprehensive Review of Intravenous Fentanyl, Butorphanol, and Midazolam in Adult and Pediatric Populations

**DOI:** 10.7759/cureus.58593

**Published:** 2024-04-19

**Authors:** Neeta Verma, Janhavi S Dahake

**Affiliations:** 1 Anesthesiology, Jawaharlal Nehru Medical College, Datta Meghe Institute of Higher Education & Research, Wardha, IND

**Keywords:** adult populations, midazolam, butorphanol, intravenous fentanyl, sedation strategies, magnetic resonance imaging (mri)

## Abstract

Magnetic resonance imaging (MRI) is a critical diagnostic tool that often requires patient sedation to ensure optimal image quality and patient comfort, particularly in those with anxiety or an inability to remain still. This comprehensive review examines the efficacy, safety, and practical considerations of three commonly used intravenous sedatives, namely, fentanyl, butorphanol, and midazolam, in adult populations undergoing MRI procedures. This review highlights the pharmacological profiles, advantages, and limitations associated with each sedative agent through a detailed analysis of current literature, clinical guidelines, and practice-based evidence. Fentanyl is noted for its potent analgesic properties and rapid onset of action, making it suitable for painful procedures. Butorphanol, with its unique opioid agonist-antagonist activity, presents an alternative with a balance between analgesia and sedation, potentially offering a safer profile for certain patient populations. Midazolam, widely recognized for its anxiolytic and amnestic effects, remains a staple in managing procedure-related anxiety. The review further discusses patient selection criteria, dosing strategies, and the importance of individualized sedation planning to enhance patient experience and procedural outcomes. Future directions highlight the potential of emerging sedation agents and non-pharmacological approaches to improve patient comfort and compliance. The findings underscore the necessity for healthcare providers to adapt sedation practices to the specific needs of each patient, considering both the clinical context and the inherent characteristics of the sedative agents. This review aims to guide clinicians in selecting the most appropriate sedation strategy for adult patients undergoing MRI, optimizing patient care and diagnostic efficacy.

## Introduction and background

Magnetic resonance imaging (MRI) is a valuable diagnostic tool utilized across various medical specialties for its ability to provide detailed images of internal body structures [1]. However, confined space, loud noises, and the requirement for patients to remain still during the procedure can induce anxiety and discomfort, leading to suboptimal image quality and compromised patient experience. Sedation is commonly employed to alleviate patient apprehension and facilitate cooperation during MRI scans [2]. Effective sedation enhances patient comfort and cooperation during MRI examinations [3]. By reducing anxiety and promoting relaxation, sedatives help minimize motion artifacts and improve image quality, thereby aiding in accurate diagnosis and treatment planning. In addition, sedation contributes to a more positive overall patient experience, potentially increasing compliance with future imaging studies [4].

Intravenous fentanyl, butorphanol, and midazolam are among the commonly used sedatives for MRI procedures [5]. Each medication possesses distinct pharmacological properties and mechanisms of action that influence its sedative effects and side effect profile. Fentanyl is a potent opioid analgesic that provides rapid onset and short duration of sedation. Butorphanol is a synthetic opioid agonist-antagonist with both analgesic and sedative properties. Midazolam belongs to the benzodiazepine class of sedatives and exerts anxiolytic, sedative, and amnestic effects [6]. The primary objective of this review is to critically evaluate and compare the efficacy, safety, and practical considerations associated with the use of intravenous fentanyl, butorphanol, and midazolam for sedation during MRI procedures in adult populations. By synthesizing existing evidence and clinical experience, this review aims to provide insights into optimal sedation strategies that optimize patient comfort, ensure image quality, and minimize procedural risks.

## Review

Intravenous fentanyl

Mechanism of Action and Pharmacokinetics

Fentanyl, a potent opioid analgesic, operates through a specific mechanism of action and showcases distinctive pharmacokinetic properties. The drug functions by binding to opioid receptors, particularly the mu-opioid receptor, which is associated with G-proteins. This binding initiates a series of events leading to the suppression of adenylate cyclase, ultimately diminishing the levels of cyclic adenosine monophosphate (cAMP) [7,8]. This mechanism yields robust analgesic effects, with a duration of action lasting several hours and a broader therapeutic index attributed to the development of tolerance to opioids. In terms of pharmacokinetics, fentanyl undergoes hepatic metabolism primarily via the CYP450 enzyme system, specifically CYP3A4. The drug experiences a high first-pass clearance and excretes approximately 75% of an intravenous dose in urine, mainly as metabolites, with less than 10% representing the unchanged drug. Roughly 9% of the dose is eliminated in the feces, predominantly as metabolites [8,9]. Fentanyl displays a three-compartment model for its pharmacokinetics, with distribution into skeletal muscle and fat, followed by gradual release into the blood. The drug exhibits a volume of distribution of 4 L/kg, a terminal elimination half-life of 219 minutes, and a redistribution time of 13 minutes. Grasping the mechanism of action and pharmacokinetic profile of fentanyl is imperative for optimizing its clinical application, ensuring accurate dosing, and effectively managing its effects across various medical scenarios.

Efficacy in Achieving Sedation During MRI

Extensive research has delved into the efficacy of achieving sedation during MRI procedures, focusing on safety, effectiveness, and nursing impact. Studies indicate that conscious sedation for MRI is safe and highly effective, boasting a success rate of 93.5% in completing diagnostic and MR examinations among adult populations [10]. Rare complications, such as oxygen desaturation, underscore the critical importance of vigilant monitoring during sedation procedures [10]. In pediatric populations, investigations have compared various soothing agents, such as propofol and pentobarbital/midazolam/fentanyl regimens. These studies highlight that propofol, with its faster onset, recovery time, and comparable efficacy, stands out as a promising alternative to traditional sedation regimens for children undergoing MRI procedures [11]. Such findings underscore the significance of selecting sedative agents that achieve optimal sedation levels and ensure swift recovery and minimal adverse events in pediatric patients. Moreover, efforts to conduct MRI in infants and young children without sedation have gained traction, emphasizing the importance of minimizing anesthesia usage whenever feasible and shortening the duration of sedated MRI exams to reduce the administered dose of anesthetic medications [12]. Techniques like MRI simulation, awake and relaxed protocols, and non-sedated imaging modifications have been deployed to enhance the success of MRI procedures without sedation in pediatric patients, underscoring the importance of meticulous planning and patient preparation [12]. Overall, the body of research underscores the efficacy of various sedation strategies in achieving successful sedation during MRI procedures across both adult and pediatric populations. The emphasis lies on prioritizing safety, effectiveness, and optimizing patient outcomes while simultaneously striving to minimize the necessity for sedation whenever feasible.

Safety Profile and Potential Adverse Effects

The safety profile and potential adverse effects of intravenous fentanyl are critical considerations in its clinical utilization. Extensive research underscores fentanyl's role as a potent synthetic opioid analgesic with significant activity in alleviating pain by binding to various sites within the central nervous system. However, akin to other narcotic opioids, fentanyl carries a spectrum of adverse effects that warrant careful attention. Adverse effects associated with fentanyl encompass a wide range, including but not limited to addiction, confusion, respiratory depression, drowsiness, nausea, visual disturbances, hallucinations, constipation, hypotension, and, in severe cases, even death [13]. Furthermore, the concurrent use of fentanyl with alcohol or other drugs like cocaine and heroin can potentiate its adverse reactions, leading to synergistic effects that exacerbate risks [13]. Of particular concern is fentanyl's propensity to induce respiratory depression, which, if untreated, can progress to respiratory arrest. This highlights the paramount importance of vigilant monitoring and precise dosing when administering intravenous fentanyl [14]. Moreover, the medication's potency and rapid onset of action pose inherent risks, mainly when dosing errors occur. Studies have identified common medication errors associated with intravenous fentanyl, including wrong-dose/overdosage events and wrong-drug events. These findings underscore the necessity for stringent protocols, double checks before administration, and standardized pain management procedures to mitigate these risks and enhance patient safety [15]. While intravenous fentanyl remains a valuable analgesic agent in pain management and anesthesia, its safety profile mandates meticulous attention to dosing, vigilant monitoring for adverse effects, and the implementation of robust risk reduction strategies in clinical practice. By adhering to stringent protocols and incorporating comprehensive safety measures, healthcare professionals can mitigate the potential for medication errors and adverse events, thereby ensuring the safe and effective use of intravenous fentanyl in patient care.

Dosage Considerations and Titration Protocols

Premedication typically involves the intravenous administration of 50 to 100 mcg (0.05 to 0.1 mg) of fentanyl, administered 30 to 60 minutes before surgery, as commonly practiced [16]. As an adjunct to general anesthesia, a recommended dosage of 50 to 100 mcg (0.05 to 0.1 mg) of fentanyl can be administered via intramuscular or slow intravenous route over one to two minutes for additional analgesia [17]. In postoperative settings, pain control in the recovery room often involves the intramuscular administration of 50 to 100 mcg (0.05 to 0.1 mg) of fentanyl, with the option for repeat doses as necessary [16]; for pediatric patients aged two to 12 years, reduced doses as low as 2 to 3 mcg/kg are recommended for induction and maintenance [16]. Intravenous fentanyl can be effectively titrated for severe cancer pain using a titration protocol, achieving pain control in approximately 11 minutes without significant adverse effects [18]. Moreover, for patient-controlled analgesia (PCA), intravenous fentanyl can be administered at 10 to 20 mcg every six to 20 minutes as needed, with initiation at the lowest effective dose and adherence to institutional protocols [8]. These dosing guidelines and titration protocols are designed to ensure adequate pain management while minimizing adverse effects, particularly in critical care and cancer pain scenarios. Individualized dosing and vigilant monitoring play pivotal roles in optimizing the benefits of intravenous fentanyl while mitigating the risks associated with opioid use.

Comparative Analysis With Other Sedatives

A comparative study examined the efficacy of using propofol alone versus a combination of propofol with midazolam for pediatric MRI sedation. Findings revealed that while the combination reduced the frequency of adverse events, it slightly prolonged the recovery time compared to propofol alone [19]. Incorporating a small dose of midazolam alongside propofol during pediatric MRI sedation has diminished adverse events, albeit at the expense of a more extended recovery profile [19]. In the realm of pediatric sedation for MRI, dexmedetomidine and propofol are frequently employed sedatives. Dexmedetomidine, an α2-adrenergic receptor agonist, and propofol, a highly effective soothing agent, are both renowned for their utility in this context [20]. Research indicates that propofol offers a shorter recovery time than dexmedetomidine, rendering it a preferred choice for pediatric sedation during MRI procedures [20].

Nonpharmacological interventions have gained traction as alternative approaches to diminish the necessity for sedation and general anesthesia in pediatric MRI settings. These interventions encompass preparation, distraction, and acknowledgment, demonstrating promising results in reducing sedation requirements, particularly among children aged three to 10 years [21]. Notably, the success rate of MRI procedures without sedation or general anesthesia significantly increased with nonpharmacological interventions, underscoring their efficacy in minimizing sedation needs in pediatric imaging contexts [21]. In assessing sedative options for pediatric MRI sedation, it is imperative to consider factors like adverse events, recovery time, and success rates. Each sedative presents its unique advantages and considerations. Hence, the selection of a sedation strategy should be tailored to individual patient needs and safety profiles. By weighing these factors carefully, healthcare providers can optimize the sedation approach for pediatric patients undergoing MRI procedures, ensuring both efficacy and safety.

Butorphanol

Pharmacological Characteristics and Mode of Action

Butorphanol, a synthetic opioid agonist-antagonist, possesses distinctive pharmacological properties and operates through a specific mode of action [22]. Acting as a partial agonist and antagonist at the μ-opioid receptor and as a partial agonist at the κ-opioid receptor, butorphanol triggers intracellular inhibition of adenylate cyclase, closure of calcium channels, and opening of potassium channels in neurons within the central nervous system [23]. These mechanisms lead to the hyperpolarization of cell membranes, inhibiting action potential transmission and providing analgesic effects [23]. Given its κ-agonist activity, butorphanol can elevate pulmonary arterial pressure and cardiac work, potentially inducing dysphoria at therapeutic or higher doses. This attribute contributes to its lower abuse potential when compared to other opioid medications. Common side effects associated with butorphanol include sedation, confusion, dizziness, nausea, vomiting, and increased perspiration, typical of opioid analgesics [24]. Furthermore, research indicates that butorphanol exhibits a lower likelihood of both physical and psychological dependency compared to other opioids like morphine, fentanyl, and oxycodone. Classified as a schedule IV drug due to its medical usefulness and low potential for abuse, butorphanol stands out for its reduced risk of dependency. Butorphanol's pharmacological profile, characterized by its dual action at μ- and κ-opioid receptors, results in analgesic effects while also influencing cardiovascular parameters and the potential for dysphoria. These unique attributes contribute to its clinical utility in pain management, offering a reduced risk of abuse and dependency relative to other opioids [25].

Clinical Evidence Regarding Sedation Efficacy in MRI

Clinical evidence regarding sedation efficacy in MRI underscores the safety and effectiveness of conscious sedation for adult populations undergoing MRI procedures. Studies have consistently demonstrated the safety of conscious sedation, with a high success rate in completing MRI examinations and minimal complications, primarily centered around oxygen desaturation [10]. Moreover, employing specialized nursing staff proficient in sedation techniques has reduced procedural variability and associated costs related to MRI sedation [10]. In pediatric populations, research highlights the importance of minimizing sedation whenever feasible to mitigate anesthesia's financial and operational impacts and the potential adverse effects of anesthetic medications [12]. Various techniques have been developed to facilitate non-sedation MRI in children, including MRI simulation for exam preparation, "asleep but not sedated" techniques, awake and relaxed approaches with certified child life specialists, animal-assisted therapy, and modifications to MRI protocols aimed at reducing motion artifact and noise [12,26].

Adverse Effects and Safety Profile

Common side effects of butorphanol encompass nausea, vomiting, drowsiness, dizziness, dry mouth, and warmth or redness under the skin [27,28]. On the other hand, serious side effects that necessitate immediate medical attention include noisy breathing, shallow breathing, slow heart rate, weak pulse, fast or pounding heartbeats, issues with urination, confusion, and lightheadedness [27,28]. In addition, rare side effects may manifest as sweating, unusual tiredness, weakness, and less common effects, such as hallucinations, fever, muscle stiffness, and diarrhea [27,28]. In terms of long-term effects, butorphanol poses potential risks associated with drug abuse, emotional lability, and head injury [22]. Butorphanol is contraindicated in cases of opioid dependence, respiratory depression, and respiratory failure, with relative contraindications observed in patients with cardiac arrhythmias, cardiovascular disease, and other relevant conditions [22]. Notably, the drug carries the potential for addiction, abuse, and misuse, leading to overdose and serious health risks [27]. Patients with specific medical conditions, such as asthma, breathing problems, liver or kidney disease, head injury, heart disease, high blood pressure, or alcoholism, should exercise caution when using butorphanol [27]. Furthermore, the drug interacts with various medications, including alvimopan, benzodiazepines, and other CNS depressants, emphasizing the necessity for monitoring and avoidance of certain combinations [27,28].

Comparison With Fentanyl and Midazolam

When comparing the use of fentanyl and midazolam, research suggests that employing fentanyl alongside low-dose midazolam leads to significantly faster recovery from sedation compared to meperidine, with no apparent disparities in sedation quality during colonoscopy procedures [29]. Similarly, a study investigating sedation options for children undergoing endoscopic procedures found that while the midazolam-ketamine combination offered effective sedation, the fentanyl-propofol combination resulted in a shorter recovery time and fewer side effects during the recovery period [30]. Furthermore, in outpatient colonoscopy settings, propofol demonstrated shorter sedation and recovery times, along with superior post-procedure neuropsychologic function when compared to the midazolam-fentanyl combination [31]. These findings suggest that propofol may confer advantages over the midazolam-fentanyl combination in specific medical procedures. Overall, these studies underscore the differences in efficacy, recovery times, and side effect profiles between fentanyl and midazolam when utilized in various sedation combinations for different medical procedures. This emphasizes the importance of selecting the most appropriate sedation strategy based on the specific requirements of the procedure and patient population.

Midazolam

Pharmacodynamics and Pharmacokinetics

Understanding the pharmacodynamics and pharmacokinetics of sedative agents is integral to comprehending their actions and effects in medical procedures. Pharmacodynamics refers to how drugs interact with the body to produce their effects. At the same time, pharmacokinetics involves studying how drugs move through the body, encompassing absorption, distribution, metabolism, and excretion [32,33]. When it comes to sedative drugs like midazolam, propofol, ketamine, and sevoflurane, it is imperative to consider their pharmacokinetic and pharmacodynamic characteristics to ensure safe and effective sedation during procedures. These sedative drugs are valued for their rapid onset of action, quick recovery, and ease of administration and monitoring, rendering them advantageous options for sedation across various medical settings. Nonetheless, each drug possesses a distinct pharmacokinetic profile that influences onset time, duration of action, and potential side effects, such as accumulation, leading to prolonged sedation or residual effects [33]. Understanding the pharmacodynamics of sedative agents is crucial for achieving the desired level of sedation while upholding patient safety and comfort. The complexity of sedation management is further underscored by considerations, such as drug interactions, synergistic effects, and variations in individual responses to sedatives, emphasizing the need to tailor drug regimens to individual patient needs. In addition, monitoring sedation levels, patient satisfaction, and striking a balance in the depth of sedation to mitigate the risk of respiratory depression are pivotal aspects of sedation practices [33,34]. By integrating knowledge of pharmacodynamics and pharmacokinetics into sedation protocols, healthcare providers can optimize sedation outcomes while prioritizing patient safety and comfort.

Safety Considerations and Common Adverse Effects

Sedatives pose various safety considerations that healthcare providers must address. Sedatives can be habit-forming and carry the potential for addiction, potentially leading to substance use disorder if misused. Healthcare professionals prescribe sedatives cautiously, emphasizing the importance of adhering to prescribed dosages to prevent adverse effects and overdose. Furthermore, different levels of sedation exist, ranging from conscious sedation to general anesthesia, each with specific uses and considerations for patient safety [35]. Proper monitoring of vital signs, alertness, coordination, and behavior is essential during and after sedation to ensure patient safety and well-being [36]. Several adverse effects are associated with the use of sedatives. First, sedation can induce decreased alertness, resulting in sleepiness and impaired judgment, affecting cognitive and motor functions. In addition, some individuals may experience agitation, restlessness, or unpredictable behavior as a result of sedation. Sedatives may exacerbate conditions, such as gastroesophageal reflux disease (GERD), and can lead to nausea or vomiting [36]. Moreover, the use of sedatives, particularly when combined with other central nervous system depressants, can heighten the risk of respiratory depression and airway obstruction [37]. Benzodiazepines, a common group of sedatives, are known to cause memory and cognitive impairment, including impaired memory, attention, learning, and cognitive abilities, potentially resulting in significant morbidity and mortality [38].

Comparative analysis with fentanyl and butorphanol

The comparative analysis between fentanyl and butorphanol provides valuable insights into their efficacy and side effects across different medical contexts. Studies suggest that fentanyl exhibits greater effectiveness and acts more rapidly in managing perioperative shivering compared to butorphanol [39]. In a study comparing the two medications for alleviating postoperative shivering in spinal anesthesia, fentanyl showed swifter relief of shivering than butorphanol, with a higher success rate in controlling the condition [40]. However, it is important to note that some patients who received fentanyl experienced shivering relapse within 30 minutes, while those administered butorphanol relapsed within 20-45 minutes, necessitating additional treatment [39]. Furthermore, the levels of sedation varied between the two medications, with more patients in the butorphanol group experiencing Grade III sedation compared to Grade II sedation in the fentanyl group [40]. Common side effects, such as sedation, nausea, vomiting, and recurrence of shivering, were reported to be more prevalent with butorphanol than with fentanyl [40]. In addition, in the context of gastrointestinal endoscopy sedation, butorphanol was compared with sufentanil, indicating that butorphanol may diminish postoperative nausea and vomiting, enhance postoperative analgesia, and decrease adverse effects compared to sufentanil [41]. While fentanyl appears to be more effective in managing perioperative shivering and may offer advantages in certain situations, butorphanol also demonstrates efficacy with potential benefits in reducing side effects, such as nausea and vomiting. The specific needs of the patient should guide the decision between fentanyl and butorphanol, the nature of the procedure, and considerations regarding effectiveness and side effect profiles.

Comparative analysis

Efficacy: Comparative Effectiveness in Achieving Adequate Sedation Levels

The comparative effectiveness of sedative medications in achieving optimal sedation levels for adult MRI procedures has undergone thorough investigation. Studies comparing propofol and dexmedetomidine for sedation in claustrophobic adults undergoing MRI have revealed that both drugs effectively reduce anxiety levels. However, dexmedetomidine may require more time to achieve sufficient anxiolysis and induce sleep compared to propofol. It is crucial to note that dexmedetomidine is associated with common adverse effects like hypotension and bradycardia, which are significant factors to consider when selecting a sedative medication [42]. Despite the availability of traditional drugs like midazolam and ketamine for sedation during MRI, they exhibit lower sedation success rates, prolonged recovery times, and significant adverse events [3]. By contrast, newer medications, such as dexmedetomidine, propofol, and sevoflurane, are preferred choices for sedation/anesthesia in both adult and pediatric MRI procedures due to their effectiveness, rapid recovery, and milder properties [3]. When aiming to achieve optimal sedation levels, the selection of the appropriate medication is paramount. Dexmedetomidine may offer advantages in preserving respiratory drive, whereas propofol is renowned for its high efficacy and swift recovery [3]. Nevertheless, the choice of sedative medication hinges on various factors, including the patient's condition, desired sedation level, recovery duration, and potential adverse effects. The comparative analysis of sedative medications for MRI sedation in adult populations underscores the necessity of balancing efficacy, safety, recovery time, and patient comfort. This ensures successful imaging procedures while prioritizing patient well-being and the quality of imaging results.

Safety: Comparison of Adverse Effect Profiles and Risk Mitigation Strategies

The safety assessment of sedation strategies for MRI in adults involves evaluating the adverse effect profiles and implementing risk mitigation strategies associated with various medications, such as propofol, dexmedetomidine, midazolam, and ketamine. Studies indicate that traditional drugs like midazolam and ketamine, while convenient to administer, exhibit lower sedation success rates, prolonged recovery times, and significant adverse events. This underscores the necessity for newer medications with enhanced safety profiles [3,21]. Dexmedetomidine, for instance, is linked to the preservation of respiratory drive, and propofol is recognized for its effectiveness and swift recovery. At the same time, sevoflurane is noted for its mild and nonirritating nature, rendering it a safer option for sedation/anesthesia during MRI procedures [21]. The transition toward safer sedation medications like dexmedetomidine, propofol, and sevoflurane, coupled with the implementation of nonpharmacological interventions, plays a pivotal role in augmenting patient safety, reducing adverse effects, and enhancing the overall experience of individuals undergoing MRI procedures.

Patient Experience and Satisfaction

Patient experience and satisfaction are paramount in outpatient imaging, particularly MRI procedures. Extensive research has demonstrated a robust correlation between patient satisfaction and outpatient MRI volumes, indicating that heightened satisfaction levels lead to increased imaging volumes [43]. This association underscores the pivotal role of patient-centered care and positive experiences in shaping healthcare utilization and operational outcomes. In procedural sedation for MRI, patient satisfaction is profoundly influenced by several factors, including comfort, anxiety alleviation, and the overall experience throughout the imaging procedure. Studies have underscored the significance of nonpharmacological interventions, such as preparation, distraction, and acknowledgment, in diminishing the necessity for sedation and general anesthesia in pediatric patients undergoing MRI [21]. These interventions augment patient comfort and contribute to favorable imaging outcomes by minimizing reliance on sedation and anesthesia. Furthermore, the choice of sedation medication, the proficiency of healthcare providers, and the overall perioperative experience substantially impact patient satisfaction during and after procedural sedation for various medical procedures, including MRI [44]. Ensuring patient comfort, safety, and favorable outcomes through efficacious sedation strategies is indispensable for enhancing patient experience and satisfaction in healthcare settings. Patient experience and satisfaction are integral components of healthcare delivery, exerting profound influences on patient outcomes, service utilization, and operational efficacy in outpatient imaging facilities, particularly those offering MRI services. Prioritizing patient-centered care, implementing nonpharmacological interventions, and optimizing sedation strategies are pivotal in elevating patient satisfaction and fostering positive healthcare experiences.

Practical Considerations

Traditional drugs, such as midazolam and ketamine, remain in use for sedation during MRI procedures owing to their ease of administration, despite potential drawbacks like low sedation success rates and prolonged recovery times [3]. Conversely, newer medications like dexmedetomidine, propofol, and sevoflurane are favored choices for sedation/anesthesia in both pediatric and adult MRI settings due to their effectiveness, rapid recovery profiles, and milder properties [3]. Anesthesia administration for MRI entails a multidisciplinary team of healthcare professionals, including anesthesiologists, nurses, and technologists, dedicated to ensuring patient safety and comfort throughout the procedure and subsequent recovery period [3]. The selection of sedative/anesthetic agents for MRI in adults involves careful consideration of the financial implications of MRI workflow and the potential adverse effects of anesthetic medications [3]. This underscores the importance of conducting MRI examinations without sedation whenever feasible, aiming to minimize costs and operational challenges. Dexmedetomidine, propofol, and sevoflurane emerge as preferred options for sedation/anesthesia in both pediatric and adult MRI scenarios due to their effectiveness and swift recovery, potentially yielding cost savings by reducing the need for prolonged recovery times and additional monitoring measures [3].

Recommendations for Sedation Strategy Selection Based on Specific Clinical Scenarios and Patient Characteristics

Children's suitability for MRI procedures without sedation depends on age and medical condition. Infants under six months old often tolerate MRI scans using asleep but not sedated techniques, while those older than six years may manage exams without sedation. However, children aged between six months and six years, particularly those with developmental delays or neuromuscular conditions, may necessitate anesthesia for MRI due to challenges in following instructions and remaining motionless during the scan [12]. Effective preparation and coordination are paramount when planning MRI exams for pediatric patients. Identifying potential candidates for non-sedated procedures, offering age-appropriate preparatory measures, and collaborating with a multidisciplinary team comprising child life specialists, nurses, technologists, and anesthesiologists are vital to ensure a smooth imaging experience [12]. Conscious sedation is safe and effective for MRI procedures, boasting a high success rate in completing diagnostic and comprehensive MR examinations. The involvement of specialized nursing staff proficient in MR sedation can mitigate procedure variability and costs, underscoring the importance of a highly specialized team in ensuring the safety and efficacy of sedation procedures during MRI [10].

Future directions

Emerging Sedation Agents and Alternative Approaches

**Figure 1:**
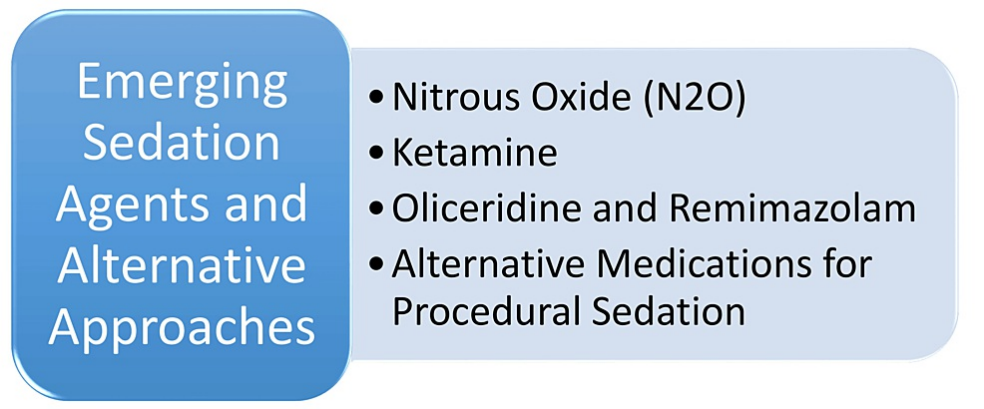
Emerging sedation agents and alternative approaches Image Credit: Dr. Janhavi S. Dahake

Nitrous oxide (N_2_O) is an inhalation gas renowned for its analgesic, anxiolytic, and anesthetic properties, making it particularly suitable for patients with needle phobia or mild to moderate anxiety. Administered via inhalation, N_2_O boasts rapid onset and clearance, facilitating safe sedation and prompt recovery post-procedure. Its flexibility allows for a combination of oxygen and titration according to individual patient response, thus delivering effective anxiolysis and analgesia within mild to moderate sedation [45]. Ketamine, a dissociative anesthetic agent, is widely employed for dental sedation, functioning by inhibiting the release of the NMDA neurotransmitter glutamine. Distinguished by its unique mechanism of action, ketamine finds utility in various medical settings, including dentistry, owing to its efficacy in inducing sedation [45]. Oliceridine and remimazolam emerge as promising sedative agents poised to reshape the landscape of moderate and deep sedation in medical practice. Oliceridine, a biased μ-receptor agonist, exerts minimal effects on the beta-arrestin pathway, thus offering analgesia with attenuated adverse effects, such as respiratory depression. Conversely, remimazolam, structurally akin to midazolam, exhibits rapid and predictable elimination due to an additional ester linkage, rendering it well-suited for moderate and deep sedation scenarios [46]. As drug shortages affect medications commonly employed for procedural sedation, alternative approaches are being explored. These strategies include reserving intravenous sedative agents for specific scenarios and considering selected anesthetics or sedatives as substitutes based on their adverse effect profiles. In addition, methods like combining multiple agents to minimize doses, rounding doses to conserve vials, and exploring inhaled anesthetics, such as nitric oxide, are being investigated to manage drug shortages effectively while ensuring optimal sedation practices [47]. Emerging sedation agents and alternative approaches are shown in Figure 1.

Areas for Further Research and Clinical Investigation

Future research endeavors could prioritize refining monitoring protocols during sedation for MRI procedures to bolster patient safety and procedural efficacy. This includes delving into the frequency and methodologies of monitoring vital signs, patient responsiveness, respiratory function, and hemodynamic variables to mitigate the likelihood of adverse outcomes. Further exploration into newer sedative agents, such as dexmedetomidine, propofol, and sevoflurane, is promising to enhance sedation success rates, expedite recovery time, and augment patient comfort during MRI examinations [48]. Comparing the efficacy and safety of these alternative sedatives with traditional medications like midazolam and ketamine is imperative for informed clinical decision-making. Tailored sedation strategies tailored to pediatric patients undergoing MRI procedures warrant dedicated research efforts, given their distinct requirements and challenges in maintaining stillness during scans [12]. Pioneering innovative techniques, such as MRI simulation experiences and age-appropriate preparation strategies, could enhance the efficacy of non-sedated exams for pediatric populations. The investigation of specialized perioperative anesthesia management protocols for MRI procedures, particularly in complex cases involving patients with claustrophobia, anxiety, or physical discomfort, presents an opportunity to streamline the sedation/anesthesia process and ensure optimal patient comfort and cooperation [49].

**Figure 2:**
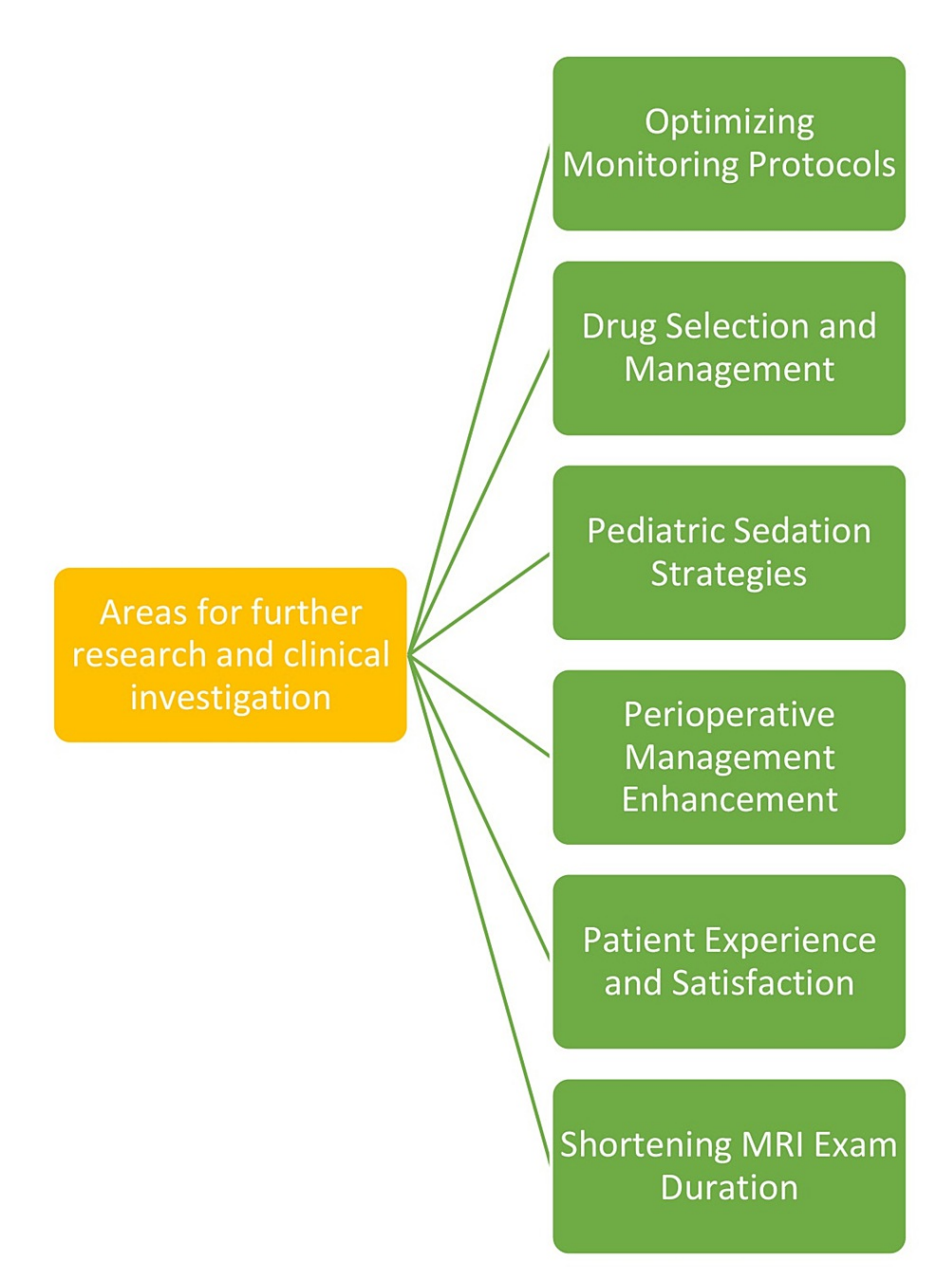
Areas for further research and clinical investigation Image credit: Dr. Janhavi S. Dahake

Further studies could investigate interventions to elevate patient experience and satisfaction during MRI procedures conducted under sedation or anesthesia. Implementing strategies to alleviate discomfort, anxiety, and sensory sensitivity, especially in patients with conditions like claustrophobia or autism, has the potential to yield superior overall outcomes and bolster patient compliance [49]. The research focused on techniques to abbreviate the duration of MRI exams, particularly for sedated patients, which is paramount to minimizing the dose of anesthetic medications administered and the associated risks [12]. Exploring methodologies, such as prospective motion correction and image reconstruction, to condense MR protocols holds promise for benefiting both sedated and non-sedated patients. Areas for further research and clinical investigation are shown in Figure 2.

## Conclusions

The comprehensive review of intravenous fentanyl, butorphanol, and midazolam for sedation in MRI procedures underscores their critical roles in enhancing patient comfort, ensuring high-quality imaging, and optimizing the overall patient experience. Each sedative offers distinct advantages and considerations, making a nuanced decision tailored to individual patient needs, procedural demands, and specific clinical contexts. The evolving landscape of sedation strategies, marked by the advent of novel agents and innovative non-pharmacological approaches, highlights the ongoing need for research and development in this area. By prioritizing safety, efficacy, and patient-centered care, healthcare professionals can continue to refine sedation practices, ensuring that patients undergoing MRI receive optimal care and experience minimal discomfort and anxiety. This approach ultimately contributes to the accuracy and efficiency of diagnostic imaging, benefiting both patients and the broader healthcare system.

## References

[1] (2024). Magnetic resonance imaging (MRI). https://www.hopkinsmedicine.org/health/treatment-tests-and-therapies/magnetic-resonance-imaging-mri.

[2] Al Shanbari NM, Alobaidi SF, Alhasawi R (2023). Assessment of anxiety associated with MRI examination among the general population in the western region of Saudi Arabia. Cureus.

[3] Wang X, Liu X, Mi J (2023). Perioperative management and drug selection for sedated/anesthetized patients undergoing MRI examination: a review. Medicine (Baltimore).

[4] Viggiano MP, Giganti F, Rossi A, Di Feo D, Vagnoli L, Calcagno G, Defilippi C (2015). Impact of psychological interventions on reducing anxiety, fear and the need for sedation in children undergoing magnetic resonance imaging. Pediatr Rep.

[5] Jung SM (2020). Drug selection for sedation and general anesthesia in children undergoing ambulatory magnetic resonance imaging. Yeungnam Univ J Med.

[6] Pathan H, Williams J (2012). Basic opioid pharmacology: an update. Br J Pain.

[7] (2024). Fentanyl. https://go.drugbank.com/drugs/DB00813.

[8] Ramos-Matos CF, Bistas KG, Lopez-Ojeda W (2024). Fentanyl. StatPearls [Internet].

[9] Smith HS (2009). Opioid metabolism. Mayo Clin Proc.

[10] Bluemke DA, Breiter SN (2000). Sedation procedures in MR imaging: safety, effectiveness, and nursing effect on examinations. Radiology.

[11] Pershad J, Wan J, Anghelescu DL (2007). Comparison of propofol with pentobarbital/midazolam/fentanyl sedation for magnetic resonance imaging of the brain in children. Pediatrics.

[12] Harrington SG, Jaimes C, Weagle KM, Greer MC, Gee MS (2022). Strategies to perform magnetic resonance imaging in infants and young children without sedation. Pediatr Radiol.

[13] Moman RN, Mowery ML, Kelley B (2024). Alfentanil. StatPearls [Internet].

[14] Weathermon R, Crabb DW (1999). Alcohol and medication interactions. Alcohol Res Health.

[15] Authority PPS (2024). Authority PPS: analysis of the multiple risks involving the use of IV fentaNYL | Advisory. Pennsylvania Patient Safety Authority. https://patientsafety.pa.gov:443/ADVISORIES/Pages/201212_122.aspx.

[16] Dave NM (2019). Premedication and induction of anaesthesia in paediatric patients. Indian J Anaesth.

[17] Euasobhon P, Dej-Arkom S, Siriussawakul A, Muangman S, Sriraj W, Pattanittum P, Lumbiganon P (2016). Lidocaine for reducing propofol-induced pain on induction of anaesthesia in adults. Cochrane Database Syst Rev.

[18] Soares LGL, Martins M, Uchoa R (2003). Intravenous fentanyl for cancer pain: a ‘fast titration’ protocol for the emergency room. J Pain Symptom Manage.

[19] Kang R, Shin YH, Gil NS, Kim KY, Yeo H, Jeong JS (2017). A comparison of the use of propofol alone and propofol with midazolam for pediatric magnetic resonance imaging sedation - a retrospective cohort study. BMC Anesthesiol.

[20] Tang Y, Meng J, Zhang X, Li J, Zhou Q (2019). Comparison of dexmedetomidine with propofol as sedatives for pediatric patients undergoing magnetic resonance imaging: A meta-analysis of randomized controlled trials with trial sequential analysis. Exp Ther Med.

[21] Thestrup J, Hybschmann J, Madsen TW (2023). Nonpharmacological interventions to reduce sedation and general anesthesia in pediatric MRI: a meta-analysis. Hosp Pediatr.

[22] LiverTox LiverTox (2012). Clinical and research information on drug-induced liver injury. https://pubmed.ncbi.nlm.nih.gov/31643176/.

[23] Al-Hasani R, Bruchas MR (2011). Molecular mechanisms of opioid receptor-dependent signaling and behavior. Anesthesiology.

[24] (2024). Butorphanol: package insert. Drugs.com. https://www.drugs.com/pro/butorphanol.html.

[25] (2024). Opioid overdose. https://www.who.int/news-room/fact-sheets/detail/opioid-overdose.

[26] Kim JG, Lee HB, Jeon SB (2019). Combination of dexmedetomidine and ketamine for magnetic resonance imaging sedation. Front Neurol.

[27] (2024). Butorphanol (Stadol) - side effects, interactions, uses, dosage, warnings. EverydayHealth.com. https://www.everydayhealth.com/drugs/butorphanol-nasal.

[28] (2024). Butorphanol: side effects, uses, dosage, interactions, warnings. RxList. https://www.rxlist.com/butorphanol/generic-drug.htm.

[29] (2024). Butorphanol | VCA Animal Hospital | VCA Animal Hospitals. Vca. https://vcahospitals.com/know-your-pet/butorphanol.

[30] Akbulut UE, Saylan S, Sengu B, Akcali GE, Erturk E, Cakir M (2017). A comparison of sedation with midazolam-ketamine versus propofol-fentanyl during endoscopy in children: a randomized trial. Eur J Gastroenterol Hepatol.

[31] Ulmer B, Hansen J, Overley C (2003). Propofol versus midazolam/fentanyl for outpatient colonoscopy: administration by nurses supervised by endoscopists. Clin Gastroenterol Hepatol.

[32] Gan TJ (2006). Pharmacokinetic and pharmacodynamic characteristics of medications used for moderate sedation. Clin Pharmacokinet.

[33] Cheng XY, Lun XQ, Li HB, Zhang ZJ (2016). Butorphanol suppresses fentanyl-induced cough during general anesthesia induction: a randomized, double-blinded, placebo-controlled clinical trial. Medicine (Baltimore).

[34] Wagner BK, O'Hara DA (1997). Pharmacokinetics and pharmacodynamics of sedatives and analgesics in the treatment of agitated critically ill patients. Clin Pharmacokinet.

[35] Liou JY, Kuo IT, Chang WK, Ting CK, Tsou MY (2023). Pharmacodynamic modeling of moderate sedation and rationale for dosing using midazolam, propofol and alfentanil. BMC Pharmacol Toxicol.

[36] (2024). Sedative. Cleveland Clinic. https://my.clevelandclinic.org/health/treatments/24880-sedative.

[37] Benzoni T, Cascella M (2024). Procedural sedation. StatPearls [Internet].

[38] (2024). Seizalam, Versed (DSC) (midazolam) dosing, indications, interactions, adverse effects, and more. https://reference.medscape.com/drug/seizalam-versed-dsc-midazolam-342907.

[39] Choi YJ, Yang SW, Kwack WG, Lee JK, Lee TH, Jang JY, Chung EK (2021). Comparative safety profiles of sedatives commonly used in clinical practice: a 10-year nationwide pharmacovigilance study in Korea. Pharmaceuticals (Basel).

[40] Wellis V (1998). Practice guidelines for the MRI & MRT.

[41] (2024). Sedation, analgesia and anaesthesia in the radiology department, second edition. https://www.rcr.ac.uk/our-services/all-our-publications/clinical-radiology-publications/sedation-analgesia-and-anaesthesia-in-the-radiology-department-second-edition/.

[42] Manne VS, Gondi SR (2017). Comparison of butorphanol and fentanyl for the relief of postoperative shivering associated with spinal anesthesia. Anesth Essays Res.

[43] Zhu X, Chen L, Zheng S, Pan L (2020). Comparison of ED95 of butorphanol and sufentanil for gastrointestinal endoscopy sedation: a randomized controlled trial. BMC Anesthesiol.

[44] Lapere C, Roelofse J, Omar Y (2015). Patient satisfaction during and following procedural sedation for ambulatory surgery. South Afr J Anaesth Analg.

[45] Yang R, Zhao R, Chaudry F, Wang T, Brunton P, Khurshid Z, Ratnayake J (2024). Modern sedative agents and techniques used in dentistry for patients with special needs: a review. J Taibah Univ Med Sci.

[46] Goudra B, Mason KP (2021). Emerging approaches in intravenous moderate and deep sedation. J Clin Med.

[47] de Castro RE, Rodríguez-Rubio M, de Magalhães-Barbosa MC, Prata-Barbosa A, Holbrook J, Kamat P, Stormorken A (2022). A review of key strategies to address the shortage of analgesics and sedatives in pediatric intensive care. Front Pediatr.

[48] (2002). Practice guidelines for sedation and analgesia by non-anesthesiologists. Anesthesiology.

[49] (2024). MRI with anesthesia: what to expect. Hospital for Special Surgery. https://www.hss.edu/conditions_mri-with-anesthesia-what-to-expect.asp.

